# Effect of amino acid infusion during cesarean delivery on newborn temperature: a randomized controlled trial

**DOI:** 10.1186/s12884-021-03734-4

**Published:** 2021-03-31

**Authors:** Krishna Pokharel, Asish Subedi, Mukesh Tripathi, Binay Kumar Biswas

**Affiliations:** 1 Department of Anesthesiology and Critical Care, BP Koirala Institute of Health Sciences, Dharan, Nepal; 2 Department of Anesthesiology and Critical Care, All India Institute of Medical Sciences, Rishikesh, India; 3 Department of Anesthesiology, ESI-Post Graduate Institute of Medical Science & Research, Kolkata, India

**Keywords:** Newborn, Amino acid, Hypothermia, Cesarean delivery, Spinal anesthesia

## Abstract

**Background:**

The effect of maternal amino acid (AA) infusion before and during cesarean delivery on neonatal temperature remains unknown. We hypothesized that thermogenic effects of AA metabolism would help maintain body temperature of newborn babies and their mothers.

**Methods:**

Seventy-six parturients scheduled for elective singleton term cesarean delivery were equally randomized to receive intravenous 200 ml of AA or placebo approximately 1 h before subarachnoid block (infusion rate:100 ml/h). The primary outcome was the newborn rectal temperature at 0, 5 and 10 min after birth. The secondary outcomes included the maternal rectal temperature at six time-points: T0 = before starting study solution infusion, T1 = 30 min after starting infusion, T2 = one hour after starting infusion, T3 = during spinal block, T4 = half an hour after spinal block, T5 = at the time of birth and T6 = at the end of infusion, as well as maternal thermal discomfort and shivering episodes.

**Results:**

There were no differences in newborn temperature between the two groups at any of the time-points (intervention-time-interaction effect, *P* = 0.206). The newborn temperature (mean [95%CI] °C) at birth was 37.5 [37.43–37.66] in the AA and 37.4 [37.34–37.55] in the placebo group. It showed a significant (*P* < 0.001) downward trend at 5 and 10 min after birth (time effect) in both groups. One neonate in the AA and five in the placebo group were hypothermic (temperature < 36.5 °C) (*P* = 0.20). There was a significant difference in the maternal temperature at all time points between the two groups (Intervention-time interaction effect, *P* < 0.001). However, after adjustment for multiplicity, the difference was significant only at T6 (*P* = 0.001). The mean difference [95%CI] in temperature decline from baseline (T0) till the end of infusion (T6) between the two groups was − 0.39 [− 0.55;− 0.22] °C (*P* < 0.0001). Six mothers receiving placebo and none receiving AA developed hypothermia (temperature < 36 °C) (*P* = 0.025). Maternal thermal discomfort and shivering episodes were unaffected by AA therapy.

**Conclusions:**

Under the conditions of this study, maternal AA infusion before and during spinal anesthesia for cesarean delivery did not influence the neonatal temperature within 10 min after birth. In addition, the maternal temperature was only maintained at two hours of AA infusion.

**Trial registration:**

ClinicalTrials.government, Identifier NCT02575170. Registered on 10th April, 2015 - Retrospectively registered.

**Supplementary Information:**

The online version contains supplementary material available at 10.1186/s12884-021-03734-4.

## Background

Inadvertent perioperative hypothermia induced by spinal anesthesia for cesarean delivery is a common phenomenon that affects up to 91% of mothers [1]. It can lead to problems like coagulopathy [2, 3], increased transfusion requirement [4], surgical site infection [5], delayed drug metabolism [6], prolonged recovery [7], shivering [8] and thermal discomfort [9]. The resulting neonatal hypothermia may be associated with an increase in respiratory distress syndrome, hypoglycemia, late onset sepsis and mortality [10]. Although a correlation between neonatal hypothermia and mortality is well established, whether low temperature is causal or merely a marker of more severe pathophysiology remains unexplained [11].

By using various active warming strategies hypothermia during cesarean delivery could be reduced [12]. A recent meta-analysis demonstrated that forced air warming or fluid warming during elective cesarean delivery decreased perioperative temperature reduction and the incidence of hypothermia [13]. Since the majority of heat is lost from the skin, actively warming from the skin surface is the most effective method [9]. Forced air warming, the most commonly practiced approach, is safe, inexpensive, and easy to use [9]. Fluid warming cannot compensate for the redistribution hypothermia, and the ongoing heat loss from the skin surface and from within surgical incisions [9]. Since the existing maternal warming methods have modest efficacy even when a multimodal strategy is applied, the search for the optimal warming technique continues [1, 14].

Intravenous (i.v.) amino acid (AA) infused before and during anesthesia is known to prevent perioperative hypothermia by increasing heat production from enhanced metabolism [1] and by resetting central thermo-sensors [15, 16]. It has been administered in pregnant women to study its fetal uptake [17, 18] and in newborns to achieve positive protein balance [19, 20]. Until now, there is no published report assessing the impact of perioperative AA infusion on maternal and newborn temperature. We hypothesized that thermogenic effects of AA infused to mothers may maintain maternal as well as newborn body temperature during cesarean delivery. Thus, we aimed to assess the effect of i.v. AA therapy on newborn and maternal core body temperature during cesarean delivery.

## Methods

This was a prospective randomized controlled double-blind study with a two-arm parallel group design with 1:1 allocation ratio. This study was approved by the Institutional Ethical Review Board, B. P. Koirala Institute of Health Sciences (BPKIHS), Nepal (Ref: ACD.599/068/069) and was conducted from June 2013 to April 2016 in the obstetric operation theatre of the university hospital of BPKIHS, Nepal. After trial commencement, no changes to methods were required. The study was registered at www.clinicaltrial.gov (NCT02575170) on 10th April 2015. The reason for delayed registration was lack of awareness on the importance of prospective registration requirements for clinical trials. This manuscript adheres to the CONSORT guidelines [21]. Women scheduled for elective full term singleton cesarean delivery with spinal anesthesia were eligible. Exclusion criteria were ASA physical status >II, age < 18 years, fetal distress, premature labor, intrauterine growth restriction, known congenital malformation and any contraindication to spinal anaesthesia.

One day prior to surgery during the pre-anesthetic visit, an investigator (AS) explained the study to all patients, and obtained written informed consent. Using a computer-generated randomization table (simple randomization without stratification or blocking), the patients were randomly allocated to one of the two groups to receive either 200 ml of AA solution (Active intervention group) (*n* = 38) or an equal volume of standard Ringer’s lactate solution (Placebo comparator group) (*n* = 38). The AA solution was a balanced mixture of 18 pure crystalline AAs, eight of which were essential AAs (Alamin SN®, Albert David Limited, Kolkata, India), and placebo fluid was standard Ringer’s lactate solution (Ringer Lactate solution®, Albert David Limited, Kolkata, India). To ensure allocation concealment, the sequentially numbered, opaque sealed envelopes (SNOSE) technique was applied. An independent researcher (SK) not involved in the trial kept the allocated group number for each patient in an opaque envelope, numbered each envelope sequentially and sealed it. Later, the same researcher (SK) handed the envelopes to an anesthesia assistant (AM) not involved in the trial or anesthesia administration. Prior to the arrival of a patient in the operation theater complex, the anesthesia assistant (AM) wrote the patient details on the envelope and then opened the envelope and prepared the drug. To maintain blinding, the bottle of AA or Ringer’s lactate was wrapped with aluminum foil and secured with an opaque white tape. It was then labelled as the ‘study infusion drug’ and the randomization number with no mention of the drug name. This was done in the absence of mothers, attending anesthetists and investigators assessing the outcomes (AS, KP) making them blinded to the study group. Approximately 90 min before expected administration of spinal anesthesia, each patient was taken to a quiet room adjacent to the operating room. Standard monitoring was applied including noninvasive blood pressure, electrocardiogram and pulse oximetry. Two i.v. channels were then secured by the anesthesia assistant (AM), one for the study drug infusion and the other for administering fluids and drugs during spinal anesthesia.

Approximately 70 min prior to surgery, a lubricated rectal thermistor probe of an anesthesia monitoring system (Life Scope 8, Nihon Khoden, Tokyo, Japan) which monitored core body temperature was inserted carefully to a depth of 3 cm by an investigator (KP or AS). The probe was secured to the buttock with tape to avoid its dislodgement. After recording the baseline vital parameters and approximately one hour prior to spinal anesthesia, i.v. infusion of the study drug was started (infusion rate:100 ml/h) by the same investigator (AS or KP).

After one hour of infusion, each patient was transferred to an operating room where the ambient temperature was set at 23 °C. Throughout the time in the operating room monitoring of non-invasive blood pressure, peripheral oxygen saturation and rectal temperature were continued. All the administered fluids were maintained at room temperature. The spinal block was performed under an aseptic condition in a lateral position with 2.2 ml of 0.5% hyperbaric bupivacaine at the L3–4 or L4–5 interspace with a 25-gauge spinal needle. Pregnant women did not receive spinal opioids and no warming devices were used.

We recorded the maternal rectal temperature at six time-points: T0 (baseline) = before starting study solution infusion, T1 = 30 min after starting infusion, T2 = one hour after starting infusion, T3 = during spinal block, T4 = half an hour after spinal block, T5 = at the time of the birth of the baby and T6 = at the end of infusion. Maternal hypothermia was defined as core body temperature < 36 °C [1]. The ambient temperature of the operating room was noted at the time of the birth of the baby. At the end of the surgery, mothers were asked to report their perception of cold related thermal discomfort during spinal anesthesia which was rated on a 0–2 subjective scale: 0 = No perception, 1 = Tolerable perception, 2 = Intolerable perception. The occurrence of shivering inside the operating room was noted using a graded scale which was validated by Crossley and Mahajan: 0 = No shivering, 1 = Piloerection or peripheral vasoconstriction but no visible shivering, 2 = Muscular activity in only one muscle group, 3 = Muscular activity in more than one muscle group but not generalized shivering, 4 = Shivering involving the whole body [22]. Pethidine 20 mg i.v. was administered by the attending anesthesiologist for grade 3 or 4 shivering. The remaining decisions regarding patient management were left to the discretion of the attending anesthesiologist.

The newborn was routinely cared for and assessed by an attending pediatrician and a nurse. After birth, a nurse immediately placed the baby on a newborn table (Babytherm 8000, Dragger, Luebeck, Germany) that was pre-set at 37 °C by combined mattress and overhead radiant warming technique. A lubricated neonatal rectal thermistor probe was then inserted into the newborn by an investigator (AS or KP) for a continuous display of temperature on a monitor (Life Scope 8, Nihon Khoden, Tokyo, Japan). Servo control with a neonatal skin temperature probe was not possible as it was not functioning. Neonatal hypothermia was defined as temperature < 36.5 °C and classified according to WHO criteria as cold stress (36–36.4 °C), moderate hypothermia (32–35.9 °C) and severe hypothermia (< 32 °C) [10]. After wiping, the body of the baby was covered with a cotton cloth and head with a cotton cap.

The primary outcome was the newborn rectal temperature at 0, 5 and 10 min after birth. The secondary outcomes were changes in the maternal temperature at various time points compared to baseline and perception of cold induced discomfort and episodes of shivering in the mother. We did not change the trial outcomes after starting this study.

### Statistical analysis

An intention to treat analysis was performed using STATA version 14.0 (Stata Corporation, College Station, TX, USA). Comparison of normally distributed continuous data between the two groups was performed using Student’s t-test. For nonparametric data, the Mann-Whitney U-test was used. Categorical data was analyzed using the Pearson chi-square test or Fisher’s exact test, as appropriate. A *P*-value of < 0.05 was considered significant. For the primary outcome, repeated-measures analysis of variance was used by fitting the main effects for intervention (i.e., AA, placebo), time (0 min, 5 min and 10 min), intervention-by-time interaction and within-subject covariance structure as compound symmetry (Repeated-measures ANOVA assumes that errors are normally distributed with a constant variance). The Greenhouse–Geisser correction for the F test was used for the violation of sphericity since the variances of the differences between all possible pairs of groups were not equal. Between-group comparisons of the intervention effect for the secondary outcome (change in maternal temperature) was performed using a mixed-effects model. Fixed-effects included time of assessment of outcome measures (the temperature at baseline to T6), study-group assignment (AA or placebo), and subject enrolled in the study as a random effect. Interaction between the time of assessment of temperature and study group was also included in the model (main effects) and an unstructured variance–covariance matrix was used. To account for the multiplicity of post hoc tests at different time points of maternal temperature, we applied the Bonferroni method. An adjustment of the alpha value was made as 0.05 divided by seven (the sum of six time points and one interaction between time of assessment and study group, i.e., 0.05/7 = 0.00714).

The Pearson correlation coefficient (r) was used to determine the relationship between the maternal temperature and the newborn temperature recorded at the time of birth. No interim analysis or stopping guidelines were applied. Neither subgroup analysis nor adjusted analysis was performed.

### Sample size calculation

A previous report showed that the mean rectal temperature of newborns immediately after cesarean delivery following spinal anesthesia was 37.7 °C (with a standard deviation of 0.4) [23]. To achieve a power of 80% for detecting an expected treatment effect of 0.5 °C (with a common standard deviation of 0.75) in the primary outcome measure of newborn temperature, assuming the type 1 error of 0.05, a sample size of 36 in each group was estimated using STATA version 12.1. Hence, we considered a sample size of 38 patients in each group as adequate to compensate for dropout cases and shift from normality in the data distribution.

## Results

**Fig. 1:**
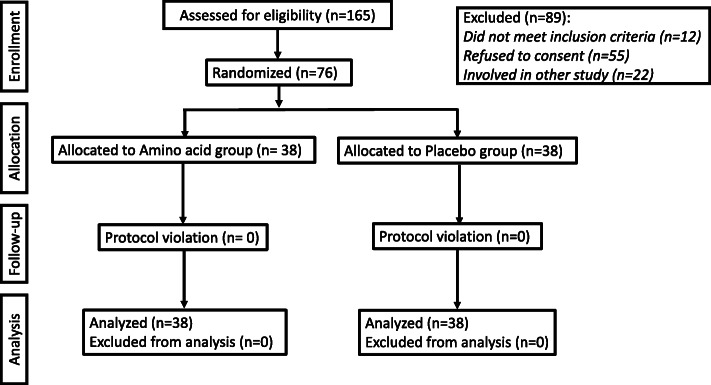
Consort flow chart

All 76 mothers completed the study protocol. The CONSORT diagram is shown in the Fig. 1 and there was no missing data. There were no differences in demographic and perioperative variables between the two groups at baseline (Table 1). Spinal anesthesia was successful in all patients. Also, newborn variables and ambient temperature at birth were comparable between the two groups (Table 2).

**Table 1:** Baseline characteristics of mothers and intraoperative variables. Values are expressed as mean (SD) or median (IQR)

	Amino acid group	Placebo group
**Age (y)**	27.2 (5.0)	28.0 (4.6)
**Weight (kg)**	60.45 (10.5)	64.0 (11.1)
**Height (cm)**	153.4 (5.9)	153.1 (5.1)
**Body Mass Index (kg/m**^**2**^**)**	25.5 (3.7)	27.2 (4.2)
**Baseline rectal temperature (°C)**	36.9 (0.27)	37.0 (0.24)
**Baseline heart rate (beats/min)**	90 (14.1)	89 (11.9)
**Baseline mean arterial pressure (mmHg)**	84 (9.1)	86 (10.4)
**Time interval from start of infusion to SAB (min)**	70.7 (5.1)	70.2 (5.4)
**Spinal block level**	T4 (T4-T5)	T4 (T4-T5)
**Intraoperative fluids (ml)**	1546 (127)	1504 (122)
**Estimated blood loss (ml)**	557 (72)	528 (60)

**Table 2:** Newborn parameters after birth. Data are presented as mean (SD), median [IQR] or number of patients

	Amino acid group	Placebo group
**Gestational age (weeks)**	39 (1.3)	39 (1.4)
**Weight (kg)**	2.8 (0.2)	2.8 (0.3)
**Ambient temperature at birth (°C)**	23.6 [0.24]	23.5 [0.21]
**Time taken to cry after birth (s)**	5 [1–15]	5 [4–10]
**APGAR score at birth**	8 [7–9]	8 [8–9]
**APGAR score at 5 min after birth**	10 [9–10]	10 [9–10]
**APGAR score at 10 min after birth**	10 [10–10]	10 [10–10]
**Suckling: well sustained/ill sustained**	35/3	36/2
**Transferred to mother side/nursery unit**	36/2	38/0

### Newborn outcomes

**Fig. 2:**
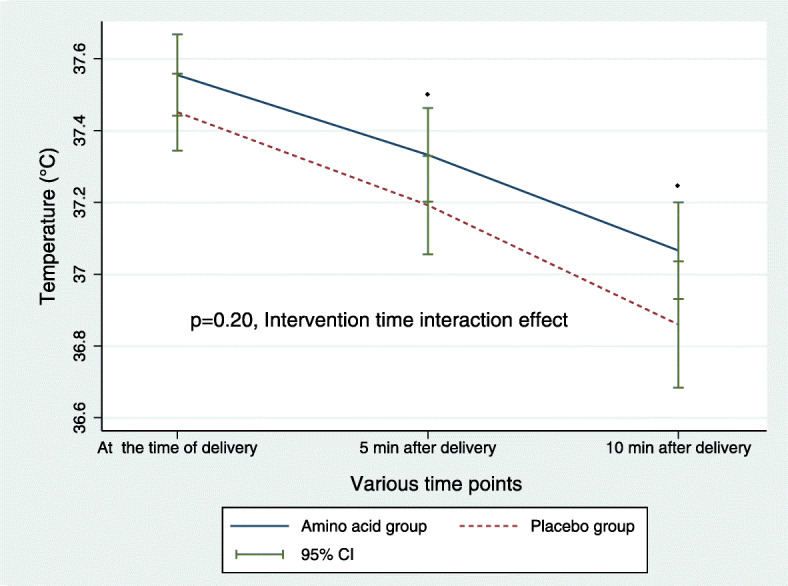
Newborn temperature (mean and CI bars) at 0, 5 and 10-min after birth. Repeated-measures ANOVA revealed a significant decline in temperature in the both groups at 5 and 10 min after birth (Time effect, ^◆^*P* < 0.001). However, there was no significant difference in temperature between the AA group and the placebo group (Intervention effect, *P* = 0.092; Intervention-time interaction effect, *P* = 0.206)

For the primary outcome (the newborn temperature at 0, 5 and 10 min after birth), repeated measures ANOVA revealed neither intervention effect (*P* = 0.092) nor intervention-time interaction effect (*P* = 0.206) (Fig. 2). Only time effect was observed because of the significant decline in postnatal temperature (*P* < 0.001) in the both groups at 5 and 10 min compared to 0 min after birth. The mean [95%CI] change in temperature (°C) from birth to 10 min was 0.48 [0.41 to 0.56] in the AA group and 0.59 [0.45 to 0.72] in the placebo group (*P* = 0.18).

At birth, none of the babies were hypothermic (< 36.5 °C). Five minutes after birth, four babies (one in the AA group and three in the placebo group) were hypothermic (cold stress, 36–36.4 °C). Ten minutes after birth, one baby in the AA group and five in the placebo group were hypothermic (*P* = 0.20). Among them, three babies in the placebo group had cold stress, two babies in the placebo group and one baby in the AA group had moderate hypothermia (32–35.9 °C). None of the babies experienced shivering.

### Maternal outcomes

**Fig. 3:**
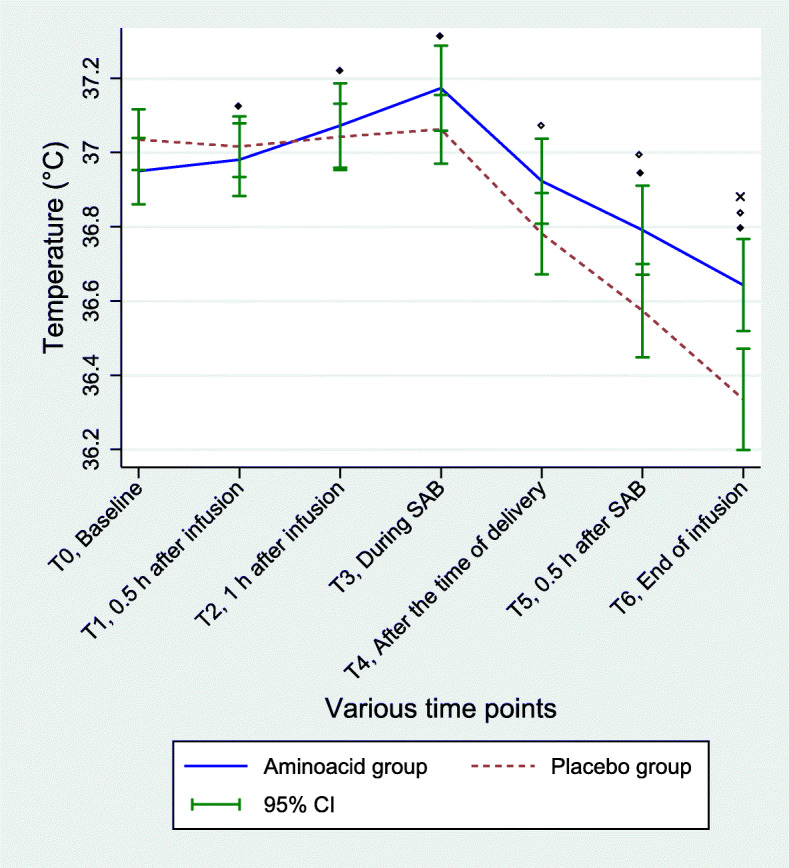
Shows the trend of maternal body temperature (mean and CI bars) at six time-points: T0 = before starting study solution infusion, T1 = 30 min after starting infusion, T2 = one hour after starting infusion, T3 = during spinal block, T4 = half an hour after spinal block, T5 = at the time of birth and T6 = at the end of infusion. The body temperature increased from baseline during AA infusion until the subarachnoid block procedure (T3). Placebo group did not show any change in body temperature till the T3 time point. After spinal anesthesia body temperature showed a steady decline in both groups, however, it remained higher in the AA group than the placebo group. Linear mixed model analysis revealed that the temperature differences from baseline in the AA group were significant at all time points except at T4 (Time effect, ^**◆**^*P* < 0.005). In the placebo group, the decline in maternal temperature from baseline was observed after spinal block at three time points T4, T5 and T6 (time effect ^**◊**^*P* < 0.005). A significant difference in temperature between the two groups was observed at all time points (Intervention effect, *P* = 0.021 at T1, *P* = 0.003 at T2, *P* < 0.001 at T3, *P* = 0.001 at T4, *P* < 0.001 at T5, and *P* < 0.001 at T6; Intervention-time interaction effect, *P* < 0.001). After adjustment for multiplicity (*P* = 0.007), the difference in the maternal temperature between the two groups was only significant at T6 (^**×**^*P* = 0.001)

Mixed model analysis revealed that mothers in the AA group had a significant change in the core body temperature from baseline at all time points except at T4 i.e., after the time of delivery. In the placebo group the time effect was observed only at T4, T5 and T6 time points. (Fig. 3). Likewise, between group differences in the maternal temperature at various time points (intervention time interaction effect) is shown in Fig. 3. The mean difference [95%CI] in temperature decline from baseline to the end of infusion between the two groups was − 0.39 [− 0.55; − 0.22] °C (*P* < 0.0001). Hypothermia (temperature < 36 °C) occurred in six mothers receiving placebo and none receiving AA therapy during spinal anaesthesia (*P* = 0.025).

Six mothers that received AA and 10 that received placebo perceived cold related discomfort (*P* = 0.540), which was intolerable in two mothers that received AA and in four mothers that received placebo. Eight (10%) patients (3 in the AA group and 5 in the placebo group, *P* = 0.430) developed shivering episodes (≥ grade 3) which responded to an i.v. meperidine 20 mg bolus.

There was a moderate positive correlation (r = 0.67, *P* < 0.001) between the maternal temperature (36.8 ± 0.34 °C) and the newborn temperature (37.5 ± 33.6 °C) at the time of delivery, with maternal temperature explaining 44% variation in newborn temperature. We did not observe any harm or unintended effects in mothers and babies of both groups.

## Discussion

In this study, we did not observe any thermogenic effect of i.v. 200 ml AA solution on neonates within 10 min after birth, when it was infused in mothers before and during scheduled cesarean delivery. Maternal core body temperature was also not influenced by the perioperative infusion of AA compared to placebo solution except at the end of two hours of therapy.

Normothermia (36.5–37.5 °C) after birth is an important goal of obstetric anesthesia [10, 11]. Trevisanuto D and colleagues (2018) have recently reported the maximum fall in newborn temperature in the first 20 min after delivery [11]. We also observed a significant fall in neonatal temperature after birth in both groups. Strategies to prevent maternal hypothermia during anaesthesia are crucial in preventing newborn hypothermia [10]. Active warming of mothers during caesarean delivery is recommended [13] to avoid morbidity related to perioperative hypothermia in new-borns [14]. Although we did not find any significant difference in the core body temperature of babies immediately after delivery in the two study groups with or without AA infusion, we did find a modest correlation between maternal temperature and neonatal temperature at birth.

The human body utilizes AA for two main purposes, namely protein accretion for growth and oxidation for energy generation [24]. In the fetus and neonate, glucose remains the principal energy substrate. At birth, the continuous trans-placental flow of glucose from the maternal circulation is terminated [25, 26]. In the first few hours, until feeding is started, energy needs are met mainly by glycogenolysis and to a lesser extent by gluconeogenesis which starts only after two hours of birth and peaks at 12 h [25, 26]. Gluconeogenesis from Alanine, an amino acid, contributes to 5–10% of hepatic glucose production on the first day of life [25]. Hence, in prenatal and the first two hours of postnatal life, AA is perhaps primarily used for protein accretion [27, 28]. The erratic as well as unpredictable transfer of AA from mother to fetus [29–31] and the minimal requirement of protein catabolism in the initial hours of birth are perhaps the main reasons behind our failure to notice any thermogenic effect of maternal i.v. AA therapy on the newborns. Maintaining ambient temperature, warming up mattresses, drying and covering the baby, and skin to skin contact of the baby with the mother [11] still remain popular techniques to prevent hypothermia in the new-borns after delivery.

Cool operating rooms and i.v. fluids maintained in ambient temperature further contribute to hypothermia [10]. During different non-obstetric surgical procedures, the thermogenic effects of AA infusion have been observed in adult patients [32–37]. We started AA infusion one-hour prior to the subarachnoid block because warming before initiation of anesthesia is the most effective approach of preventing hypothermia [10, 14]. However, we found that mothers receiving AA were only significantly warmer than those receiving placebo fluid infusion at the end of two hours of infusion.

Neuraxial anaesthesia per se is associated with hypothermia [9]. It has been correlated with impaired central thermoregulatory control and lack of responses to hypothermia such as vasoconstriction and shivering [9]. AA infusion has been reported to prevent a fall in body temperature by a mean difference of 0.5 °C at the end of 120 min of arthroplasty surgery under spinal anaesthesia [37]. Since caesarean delivery is a shorter procedure, we recorded maternal temperature until the end of AA infusion; that is up to 60 min after the spinal anaesthesia. This might be the reason for a lesser impact of AA therapy on the maternal temperature in our study (mean difference between the two groups being 0.39 °C). None of the hypothermic mothers had received AA therapy in the perioperative period, and a longer duration of temperature measurements, instead of limiting to the infusion period would probably have generated greater differences between two groups because of the ongoing effects of AA oxidation.

In this study, only 10% of the women reported thermal discomfort and 21% experienced shivering. Despite the fall in temperature patients may not experience thermal discomfort and shivering [9]. This could be because of a warm input to the central controller from the portion of the body below the level of the neural blockade [38]. Other contributory factors include impairment of central thermoregulatory control and blockade of nerves controlling vasoconstriction and shivering [9]. Interestingly, there are also reports of improved thermal comfort and decreased shivering when mothers received active warming during spinal anesthesia [13]. We also found a non-significant trend towards increased shivering and thermal discomfort in the placebo group. Based on the pooled data of multiple reports, the incidence of shivering appears to be reduced with AA therapy by a risk ratio of 0.34, but the data was highly heterogenic [36, 39].

Our study has some limitations. We accept that the core body temperature monitoring was limited to 10 min after birth for neonates and the infusion period for mothers. Hence, the extended potential effects of the ongoing metabolism of AA could not be explored. Likewise, since our study population included only healthy, fully grown term neonates, our findings cannot be extrapolated to sick, preterm or small for gestational age neonates. Furthermore, the body surface area impacts heat loss, but this data was not recorded. In addition, we were unable to measure umbilical cord blood pH and neonatal blood AA levels and the scale used to measure thermal discomfort in mothers may need further validation.

In conclusion, under the conditions of this study, amino acid infusion in mothers before and during spinal anesthesia for cesarean delivery did not influence neonatal temperature in the first 10 min of life. There was a positive effect of amino acid on maternal temperature at the end of two hours of infusion, but not before.

## Supplementary Information

**Additional file 1.**

## Data Availability

The datasets used and/or analyzed during the current study are available from the corresponding author on reasonable request. All relevant data are within the manuscript and its supporting information files.

## Abbreviations

AA: Amino acids
AM: Anantaram Majhi
ANOVA: Analysis of variance
AS: Asish Subedi
C: Celsius
CI: Confidence Interval
CONSORT: Consolidated Standards of Reporting Trials
h: hour
i.v.: intravenous
IQR: Interquartile range
kg: Kilogram
KP: Krishna Pokharel
m: meter
min: minute
mL: milli-Liter
s: second
SD: Standard deviation
SK: Sindhu Khatiwada
